# Research hotspots and trends analysis of user experience: Knowledge maps visualization and theoretical framework construction

**DOI:** 10.3389/fpsyg.2022.990663

**Published:** 2022-09-28

**Authors:** Ning Song, Xuemei He, Yin Kuang

**Affiliations:** College of Design and Art, Shaanxi University of Science and Technology, Xi’an, China

**Keywords:** user experience, knowledge map, data visualization, CiteSpace, Carrot2

## Abstract

This study focuses on user experience from the perspective of big data to complete the aggregation, clustering, and visual presentation of knowledge. Using a combination of sample literature review, visualization technologies, knowledge map analysis, Carrot2 clustering, and other methodologies, this study intends to examine user experience from three perspectives: research state, hotspots, and trends. First, based on the double-map overlay, core institutions, core countries, core authors, core journals, and core references distribution research, the knowledge flow, research power, and research subjects of user experience are analyzed. Secondly, through keyword clustering analysis, this research intuitively presents the research topics of user experience and reveals the research hotspots and the evolution path of research methods. Finally, with the help of the subject clustering algorithm, the emerging trends of user experience research are predicted: the immersive experience upgrade of multi-scenario integration, the innovative design of multi-role collaboration, and the cross-disciplinary interactive exploration of multi-discipline. Following this, the user experience knowledge map is constructed, providing a global view and macro-cognition for subsequent research.

## Introduction

User experience refers to people’s cognitive impressions and responses to the product, system, or service they use or expect to use. It includes psychological analysis, human-computer interaction, product design, emotional research, and other aspects. In the context of big data, the definition of design disciplines is constantly expanding, research paradigms and application fields are gradually extending, and user experience research is becoming progressively more precise and diversified (Xu et al., 2022). This paper used knowledge map technology to explore user experience research hotspots and future trends. By constructing a knowledge map through data visualization analysis, we can systematically and comprehensively learn the research context, knowledge system, and practice paradigm of user experience. Due to the strong interdisciplinary nature of user experience, this paper mainly focuses on three important fields of art design, computer science and management science. Based on this, the research context analysis and knowledge map construction are carried out to realize the integration of art design, science, and technology.

The literature review shows that the current domestic and foreign research on user experience is primarily the theme of theoretical analysis (Adikari et al., 2011; Wang et al., 2013; Xia and Zhang, 2013; Guo, 2016) and empirical application (Fong et al., 2015; Feng and Wei, 2019; Svezhenov et al., 2019). There are few research reviews or knowledge map literature. Integrating existing studies and knowledge map literature, the research team divides existing literature into concept sorting, domain extension, and knowledge clustering according to the research content.

In terms of concept sorting, it mainly includes the definition of user experience concepts and element analysis. For example, Shengli (2008) thoroughly explored the purpose, content, and characteristics of foreign user experience and applied the results to e-commerce, website construction, and other fields to guide the development of information services. Ding et al. (2016) reviewed the classic literature at home and abroad, systematically sorted out the concept, composition, measurement, evaluation, and application status of user experience, and analyzed the research methods of user experience. Karray et al. (2017) take human computer interaction as the theme, summarize the basic definitions, terms, and existing user experience technologies, and provide many references for interaction design applications.

The domain extension mainly focuses on developing and applying user experience in a specific field or context—for example, Bitkina et al. (2020) used the literature survey method to analyze the current medical equipment user experience situation. From the perspective of user type, medical equipment range, and use area, they discussed the primary analysis methods and advantages and disadvantages of user experience. Yuqi et al. (2019) reinterpreted the characteristics and evaluation methods of user experience based on artificial intelligence technology and further prospected the research trend of user experience driven by artificial intelligence. Vermeeren et al. (2010) summarized user experience measurement methods in the intelligent era, contributing to the realization of user experience strategy.

Regarding knowledge clustering, scholars mainly rely on data analysis and visualization software to analyze the research status and hotspots of user experience. For example, Lin et al. (2021) used keyword co-occurrence and multivariate statistical analysis methods based on the visual analysis of knowledge map to output the frontier co-word knowledge map and mainstream fields of domestic perceptual engineering research. Li et al. (2022) used bibliometrics and knowledge map analysis to analyze literature on user experience design from 1999 to 2019 and systematically reviewed the development process of user experience design from different perspectives, such as keywords, references, and author institutions. Xia and Liu (2022) used knowledge map technology to analyze the time and space of user experience journals, authors, institutions, and other information from 2006 to 2021. They obtained the differences between domestic and foreign user experience research.

The above studies are all centered on user experience and summarize information on user experience research from different perspectives, making significant contributions to the definition of discipline concepts and development path planning. However, most of the data sources of the articles are related to domestic research or describe user experience in a specific field. Furthermore, the research results lack a certain degree of systematicness, lack macro description, and research trend exploration of user experience research context. Because of this, this paper uses the Web of Science core collection database as the literature source and uses methods such as literature analysis, knowledge map, and subject clustering to objectively reveal the current research status, research subjects, and hotspots of user experience. Moreover, on this basis, construct the future development picture of user experience, and provide information support for the development and application of this field.

## Data sources and methods

### Data sources

User experience research has a wide range, involving multi-angle research on user behavior, user needs, and user emotions. To improve the diversity and intersection of data sources, this paper selects the core collection database of Web of Science, including SCI, SSCI, A&HCI, CPI-S, and other documents. Afterward, to better focus on the research field, we carried out an advanced search with “TS = (user experience OR user research) AND TS = (design)” as the search subject and selected the document types as “Article” and “Review.” The time range was from January 2011 to April 2022, and a total of 1950 articles were retrieved. Ultimately, we manually eliminated topics that deviated from user experience research and selected 1,759 documents as the primary data set.

### Research methods

**FIGURE 1:**
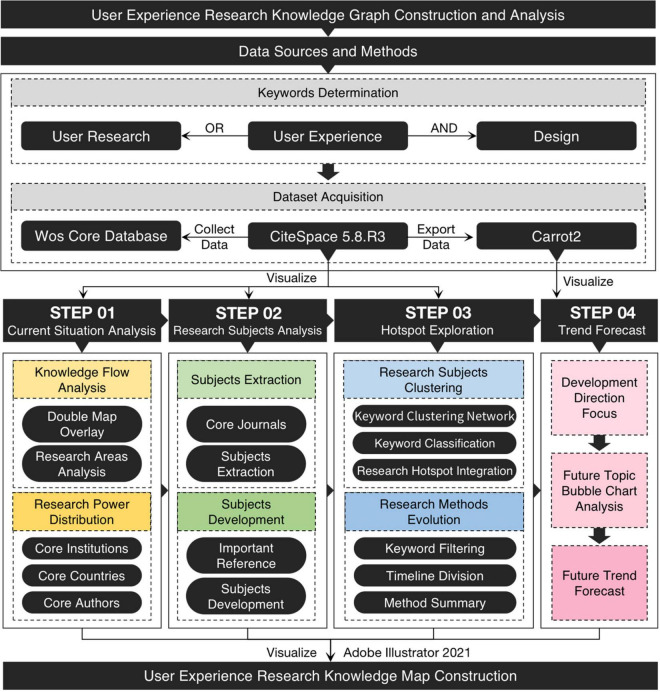
The research framework of this paper.

With the help of CiteSpace5.8.R3, Carrot2, and other software, we visually analyzed the user experience data set and constructed the user experience knowledge map by combining qualitative research and quantitative analysis. Figure 1 shows the research framework. The main steps are as follows:

1. Analyze the knowledge flow path based on the double-map overlay technology and macro-control the application field and development direction of user experience. Then, according to bibliometrics, using the knowledge map analysis method, from three perspectives of core institutions, core countries, and core authors, sort out the distribution of domestic and foreign user experience research forces.
2. Use the cluster analysis method to complete the mutation analysis of core journals and references and refine the research subjects and development context of user experience.
3. Combine the results of subject analysis, keyword clustering map, and timeline map to integrate user experience research hotspots and cutting-edge methods.
4. With the help of Carrot2 cluster analysis, we generated the topic bubble map, combined with the research status and hotspots to predict the user experience research trend, and constructed a visual knowledge map.

## Research status analysis

### Knowledge flow analysis

Knowledge flow analysis mainly explores the literature distribution, cited track, topic change, and other user experience information in different disciplines by studying the connection between cited literature and cited literature (Zhan et al., 2021). This research uses the dual-map overlay technology of CiteSpace software to analyze the knowledge flow. It uses the Blondel algorithm to form a visual journal cluster to obtain the key areas and development links of user experience research.

**FIGURE 2:**
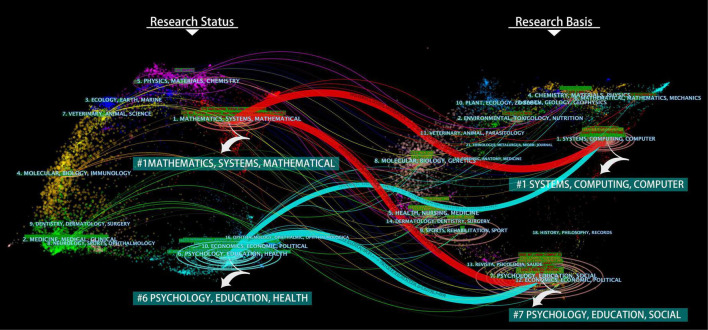
Flow trajectory of user experience core knowledge.

The dual-map overlay is divided into left and right parts. The left side of the map is the distribution of main disciplines cited in literature published, representing the research status of user experience topics. The right side of the map is the subject field of cited literature, which means the basis of user experience research (Wei and Zhang, 2020). We used the Z-Score algorithm built into the software for clustering processing of the atlas and obtained four core knowledge flow tracks, as shown in Figure 2.

As can be seen from Figure 2, user experience research mainly develops from the fields of #1 SYSTEMS, COMPUTING, COMPUTER, and #7 PSYCHOLOGY, EDUCATION, SOCIAL to #1 MATHEMATICS, SYSTEMS, MATHEMATICAL, and #6 PSYCHOLOGY, EDUCATION, HEALTH. In the research status area, the fields of #7 PSYCHOLOGY, EDUCATION, and SOCIAL are relatively complex, indicating that this field has an advantage in the number of papers and authors and is the focus and hotspot of current user experience research. Comprehensive analysis shows that the area of user experience research shows a scattered trend, covering mathematics, medicine, ecology, psychology, and other fields, reflecting the intersection and diversity of disciplines. Meanwhile, apart from the two main areas of #1 MATHEMATICS, SYSTEMS, MATHEMATICAL, and #6 PSYCHOLOGY, EDUCATION, HEALTH, user experience research is extending to #5 PHYSICS, MATERIALS, CHEMISTRY, and #2 MEDICINE, MEDICAL, CLINICAL. Moreover, with good development prospects, pay more attention to user health and ecological balance.

### Research power distribution

Institutions, countries, and authors are critical elements of academic development. Analyzing the relationship between cooperative institutions, cooperative countries, and core authors in a specific field is helpful to understanding the distribution of significant research forces quickly and provides a reference for evaluating academic achievements, introducing educational resources, and conducting international exchanges (Zhu and Hua, 2017). Based on the cooperation network analysis function of CiteSpace, this paper analyzes the cooperation network of user experience research from the three dimensions of institution, country, and author to grasp the distribution of user experience research power.

#### Core institutions

**FIGURE 3:**
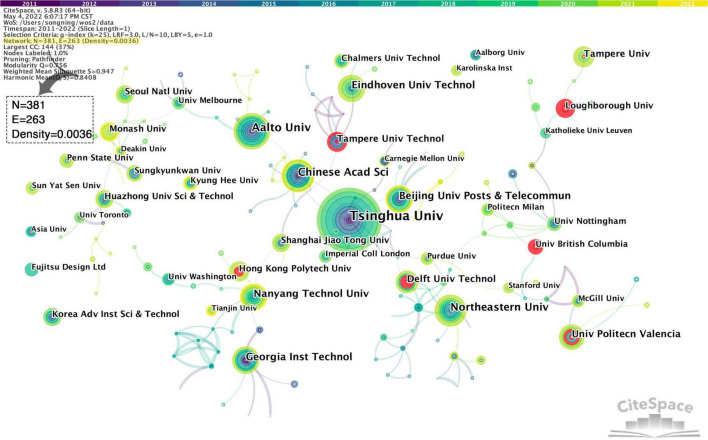
Cooperation network of core institutions.

Figure 3 shows the academic cooperation between institutions, in which the institutional cooperation network has 381 nodes and 263 connections, and the network density is 0.0036. Nodes size reflects the number of papers published, and the color of the connection between the nodes represents the year and degree of cooperation between the institutions. Through observation, Figure 3 shows a decentralized situation, which means that the cooperation between research institutions in user experience is relatively loose. The prominent research institutions are colleges and universities in various countries, and enterprises are less involved. Among them, Tsinghua University is the most prominent, representing that this institution has a leading position in the field of user experience.

Furthermore, some individual nodes are isolated on the network’s edge, such as Sun Yat-sen University, Asia University, and Fujitsu Design Co., Ltd., which shows that some research institutions have strong independence and cooperation between institutions needs improvement. Export the information of the top 10 institutions in the figure and sort out their cooperative relationships, as shown in Table 1.

**TABLE 1:** Cooperation analysis of the top 10 core institutions.

Rank	Counts	Institution	Country	Main cooperation institutions
1	34	Tsinghua University	China	Chinese Academy of Sciences, Beijing University of Posts and Telecommunications, Purdue University, etc.
2	20	Aalto University	Finland	Tsinghua University, Chinese Academy of Sciences, Tampere University of Technology, etc.
3	18	Chinese Academy of Sciences	China	Tsinghua University, Aalto University, Tampere University of Technology, etc.
4	17	Northeastern University	China	Delft University of Technology, Purdue University, etc.
5	15	Nanyang Technological University	Singapore	Hong Kong Polytechnic University, Shanghai Jiao Tong University, Tianjin University, etc.
6	15	Georgia Institute of Technology	U.S.	Nanyang Technological University
7	15	Eindhoven University of Technology	Netherlands	Tampere University of Technology, Chalmers University of Technology
8	15	Beijing University of Posts and Telecommunications	China	Tsinghua University
9	14	Valencia Polytechnic University	Spain	McGill University
10	12	Delft University of Technology	Netherlands	Northeastern University, Purdue University

According to Table 1, there is a slight difference in the number of publications among institutions, and we divided it into three types of clusters:

1. The cooperation network headed by Tsinghua University, Aalto University, and the Chinese Academy of Sciences is the most obvious, ranking the top three in terms of the number of papers and citation frequency. Furthermore, it has links with Beijing University of Posts and Telecommunications, Tampere University of Technology, Purdue University, Eindhoven University of Technology, and other institutions.

2. The network of collaborations consisting of Northeastern University, Delft University of Technology, Purdue University, and other institutions came in second. In this cooperation network, Northeastern University is the leading research institution with a high publication volume, while Purdue University is the active node. It cooperates with Tsinghua University and Northeastern University, the link between cluster 1 and cluster 2.

3. The cooperation network with Nanyang Technological University and Georgia Institute of Technology as the main, and Hong Kong Polytechnic University, Shanghai Jiao Tong University, Tianjin University, and other institutions as auxiliary institutions ranks third. Compared with the first two types of clusters, the cooperation density of cluster 3 is significantly weakened. However, more surrounding sub-nodes indicate that this cluster has been relatively active in user research in recent years and can continue to pay attention.

#### Core countries

**FIGURE 4:**
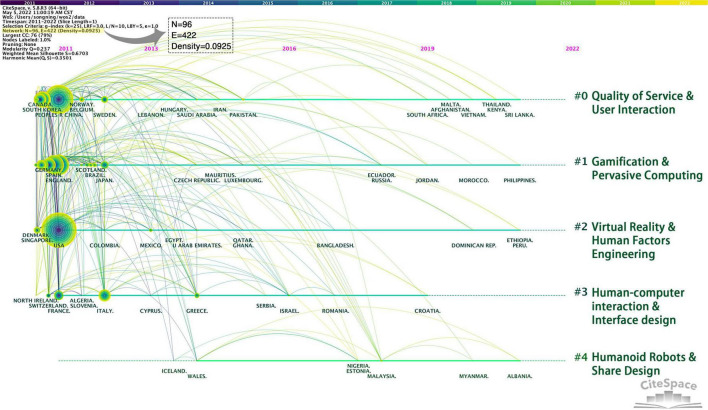
Timeline map of cooperative countries.

By analyzing the cooperative relationship between countries through the timeline diagram, it is possible to obtain the time when the relevant research topic of a specific country began and the historical span of cooperative research in different countries (Hou et al., 2018). Figure 4 is a timeline diagram of cooperative countries clustered by five keywords: Quality of Service and User Interaction, Gamification and Pervasive Computing, Virtual Reality and Human Factors Engineering, Human-computer interaction and Interface design, Humanoid Robots and Share Design. The size of the nodes represents the number of publications in the field of user experience research in different countries, and the connection between nodes represents the cooperative relationship and research flow between countries.

As shown in Figure 4, the cooperative country timeline map contains 96 nodes, 422 connections, and 5 clusters. The nodes and links in the figure are widely distributed, indicating that user experience development has been relatively scattered in recent years, and related academic research has been carried out in many countries. We further analyze the two aspects of publication volume and cooperation themes:

1. In terms of the number of published articles, user experience research is dominated by China (350 articles) and the United States (350 articles), followed by the United Kingdom (161 articles), Spain (132 articles), South Korea (115 articles), Germany (100 articles), and other countries.
2. Regarding cooperation topics, timelines #0, #1, and #2 are relatively complete, representing the main research directions of various countries in the field of user experience. In contrast, timelines #3 and #4 are relatively weak, indicating that countries’ cooperation on this topic needs to be strengthened. The #0 Quality of Service and User Interaction theme is dominated by China, South Korea, Canada, Australia, and other countries, spreading to South Africa, Malta, Thailand, and other countries in 2019. The #1 Gamification and Pervasive Computing theme research focuses on the United Kingdom, Spain, Germany, Finland, and other countries. The #2 Virtual Reality and Human Factors Engineering theme is mainly concentrated in the United States and has a small amount of cooperation with Singapore, Denmark, Mexico, and other countries. The #3 Human-computer interaction and Interface design is mainly in Italy, France, Switzerland, and other countries. After 2019, the connection disappears, indicating that research in various countries has turned to other themes. The #4 Humanoid Robots and Share Design has been published in the past 5 years. It is an emerging trend in the field of user experience. There are only a few countries, such as Malaysia, Wales, and Iceland. Cooperation between countries needs to be strengthened.

From a comprehensive analysis at the institutional and national levels, the user experience research strength is centered on China and the United States. In the Asian region, with China as the core, its research themes mainly focus on user experience theory research, user experience quality evaluation, user perception measurement, and network information security. The United States and other countries combine computer technology with user experience research and explore models and methods for combining virtual reality technology, cloud computing networks, and user experience.

#### Core authors

Authors who have many works published or who are frequently cited are considered to be “core authors” in a field. And we can use Lotka’s law to calculate their distribution (Wang and Lv, 2021). To identify the most influential groups of scholars and their cooperative relationships in the existing research, the CiteSpace software was used to generate an analysis network of co-authors. The obtained network contained 400 nodes and 187 connections, and the network density was 0.0023. Among them, Jaehyun Park, a scholar from South Korea, took the lead with ten papers. Korean scholars Hyun K Kim and Sung H Han followed with six articles. Singaporean scholar Chun-Hsien Chen, and Chinese scholar Huiyue Wu, published five related papers, and the above is the core authors in the field of user experience. According to the network connection and the ranking of authors’ published articles, on the one hand, the network layout of co-authors is relatively scattered, and the cooperation between authors is not close enough. Generally, authors from the same institution or country conduct joint research, and an extensive research team has not yet been established. On the other hand, there is little difference in the number of papers published by the authors, indicating that the research in this field is not concentrated enough, and there is a lack of expert-level scholars focusing on this field.

**FIGURE 5:**
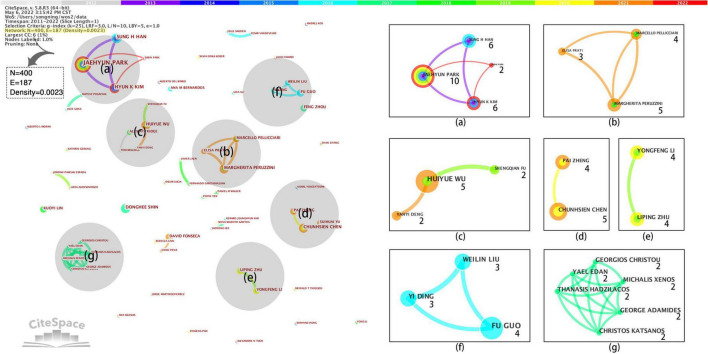
Core author network partition.

In order to further analyze the direction of cooperation among core authors, the author cooperation network is further subdivided according to the number of authors’ publications, publication time, and cooperation relationship. Seven major cooperation clusters can be captured and numbered according to the influence of core authors, as shown in Figure 5.

Figure 5 divided the author’s collaboration cluster into basic theory research, interactive technology research, and quantitative analysis research.

The first category is basic theoretical research, composed of scholars from South Korea, Singapore, China, and other countries. Among them, cluster (a) is a team led by scholar Jaehyun Park of Incheon National University in South Korea. It is the largest cluster in the cooperation network. The principal members include Hyun K Kim, Sung H Han, Subin Park, and other scholars also from South Korea (Cho et al., 2011; Park and Han, 2013). Its research subjects tend to be user experience basic theory research, including user experience framework design, method innovation, and case application. Similar to the research subject, clusters (d) are dominated by Chun-Hsien Chen scholars from Nanyang Technological University and Pai Zheng scholars from Hong Kong Polytechnic University (Huang et al., 2012; Zheng et al., 2018). However, the latter is more inclined to study the application of user experience in service system development and iteration.

The second category is interactive technology research, composed of European and Chinese scholars, focusing on interactive technology research. Among them, cluster (b) is led by Margherita Peruzzini, scholars from the University of Modena in Italy. Its members include other scholars, such as Marcello Pellicciari and Elisa Prati (Peruzzini et al., 2013, 2020). They mainly analyze the optimization and evaluation of user experience in human-computer interaction. Cluster (c) is from Sun Yat-sen University in China, led by scholars (Wu et al., 2016, 2019), to discuss the relationship between virtual reality technology and user experience.

The third category is quantitative analysis research, which is composed of Chinese scholars studying quantitative user experience methods. Among them, cluster (e) is led by Guo Fu scholars from Northeastern University (Guo et al., 2016), using eye trackers, EEG, and other experimental instruments to analyze user experience quantitatively. The cluster (f) comprises Li Yongfeng and Zhu Liping, scholars from Jiangsu Normal University (Li and Zhu, 2017, 2019), who apply fuzzy theory to user experience evaluation to realize its quantitative research.

It is worth noting that most scholars are isolated in the cooperative network. Although such authors have not established apparent collaborative relationships with other authors, they are still worthy of attention from the analysis of the number of articles published and the degree of an outbreak. For example, the Korean scholar Shin (2009, 2017, 2018, 2019), focusing on the integration of user experience design and virtual reality technology, using computer models to predict user needs and experience changes, and has achieved remarkable success in immersive interaction design. The Spanish scholar David Fonseca (Villagrasa et al., 2014) focused on the relationship between gamification design and user experience.

### Research power distribution

The theme sorting of user experience mainly includes theme extraction and theme development. Starting from the core journals and essential references, combined with the literature analysis method, we refined the development context of user experience and the direction of theme change. Furthermore, provide theoretical support for follow-up research hotspots and frontier trend analysis.

#### Theme extraction

The analysis of core journals can quickly lock high-quality information in the research field, reveal the field’s research topic and development level, and then achieve topic extraction (Yang et al., 2021). Select “Cited Journal” in the CiteSpace software to conduct a co-citation analysis of the journals, and select the top 10 core journals for research, as shown in Table 2.

**TABLE 2:** Co-citation analysis of top 10 core journals.

Rank	Counts	Journal	Main subject
1	578	Lecture notes in computer science	Computer, informatics
2	336	International journal of human-computer studies	Computer, artificial intelligence
3	276	Behavior and information technology	Informatics, behavior
4	259	International journal of human-computer interaction	Computer, interactive design
5	258	Computers in human behavior	Computer, behavior
6	246	Interacting with computers	Computer, interactive design
7	241	Communications of the acm	Computer, engineering technology
8	238	Proceedings of the sigchi conference on human factors in computing systems	Computer, ergonomics
9	197	Applied ergonomics	Engineering technology, ergonomics
10	185	Management information systems quarterly	Management, computer

It can be seen from Table 2 that the core journals related to user experience research are organized around the following three topics:

1. **Interaction design.** *International Journal of Human-Computer Interaction* and *Interacting with Computers* combines interaction design with computer technology. They are essential journals in interaction design, including cognitive, creative, social, health, and human interaction design research. The journal *Behavior* and *Information Technology*’s theme is similar to the former. However, it focuses on the connection between user experience, behavior, and informatics, emphasizing user-centered design methods. From the perspective of design research, it provides adequate methods and strategies for element selection, user research, and model building of user experience.
2. **Ergonomics.** *Proceedings of the Sigchi Conference on Human Factors in Computing Systems and Applied Ergonomics* focus on the coordination and comfort between users, machines, and systems, combining psychology, engineering, and design research to optimize system performance and improve user experience continuously. Ergonomics-centered research provides practical technical support and measurement methods for user experience research. The application of quantitative research, experimental design, and user experience analysis lays the foundation for its multidisciplinary integration.
3. **Computer science.** This topic has the highest number of citations and the most significant number of journals, including *Lecture Notes in Computer Science*, *International Journal of Human-Computer Studies*, *International Journal of Human-Computer Interaction*, and other representative journals. While these journals all focus on computer science, the emphasis varies. Among them, *Lecture Notes in Computer Science* and *Communications of the Acm* pay more attention to the research of computer algorithms, programs, information processing, and other topics, emphasizing the practical application of computer theory. *The International Journal of Human-Computer Studies* is more inclined to explore artificial intelligence technology, aiming to explore the frontier trends of computer technology. *Computers in Human Behavior* and *Management Information Systems Quarterly* combine behavior, management, and computer technology and tend to analyze and predict behavior quantitatively. With the development of computer and artificial intelligence, the integration of computer science and user experience has gradually become a hot spot and trend in user experience research, making it more precise, information-based, and intelligent.

#### Theme development

Thematic development comes from the integrated analysis of mutation citations, which is used to grasp the development trend of a specific field and map emerging thematic changes. Mutational citations are node documents that refer to sudden changes in usage. Documents located at nodes usually represent the rise or change of a particular field and are innovative and forward-looking (Rawat and Sood, 2021). We used the CiteSpace correlation algorithm to analyze the citations and obtained ten documents with the highest intensity of user experience topics in the core database of Web of Science, as shown in Table 3.

**TABLE 3:** Mutation analysis of top 10 citations.

References	Title	Strength	Begin	End	2011–2022	Subject
Law et al. (2009)	Understanding, scoping and defining user experience: a survey approach	9.35	2011	2014	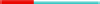	Concept definition
Desmet and Hekkert (2007)	Framework of product experience	4.04	2011	2012	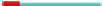	Design framework
Hassenzahl et al. (2010)	Needs, affect, and interactive products—Facets of user experience	3.7	2011	2015	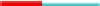	Design framework
Law and van Schaik (2010)	Modeling user experience—An agenda for research and practice	4.62	2012	2015	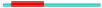	Model building
Park et al. (2013)	Modeling user experience: A case study on a mobile device	3.73	2014	2017	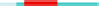	Model building
Law et al. (2014)	Attitudes toward user experience (UX) measurement	4.13	2015	2017	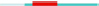	Tool development
Porat and Tractinsky (2012)	The effects of web-store design on consumers’ emotions and attitudes	3.03	2015	2016	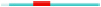	Emotional evaluation
Preece and Rogers (2015)	Interaction design: Beyond human-computer interaction	5.42	2019	2020	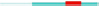	Interaction design
Schrepp et al. (2017a,b)	Construction of a benchmark for the User Experience Questionnaire (UEQ)	4.43	2019	2022	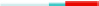	Quantitative study
Schrepp et al. (2017a,b)	Design and evaluation of a short version of the User Experience Questionnaire (UEQ-S)	3.93	2020	2022	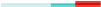	Quantitative Study

It can be seen from Table 3 that the literature with the highest emergent intensity is Understanding, scoping, and defining user experience: a survey approach by Law et al. (2009), and the research topic is the definition of user experience. Through a large number of user research, the author endows the user experience with dynamic, continuous, and subjectivity characteristics and proposes that the user experience should be a part of human-computer interaction and always follow the user-centered design principle. This document defines the nature and scope of user experience and lays the foundation for user experience research.

Subsequently, the research topic changed from concept definition to design framework research and model construction. Desmet and Hekkert (2007) and Hassenzahl et al. (2010) published a literature *Framework of Product Experience and Needs, affect, and interactive products—Facets of user experience*, respectively, according to user needs and system characteristics, the design elements are screened. Moreover, they developed a differentiated user experience framework for more accurate design optimization. Based on the framework study, Park et al. (2013) further constructed various user experience models and tried to quantify the user experience. *A Case Study on a Mobile Device* was proposed to obtain user experience data from 22 dimensions to improve the study’s accuracy.

In recent years, user experience has been gradually refined, and the research topic has shifted from macro model to micro tool or method innovation. It includes the measurement of user satisfaction (Haryaka et al., 2017), user emotion valence (Seo et al., 2015), interactive interface availability (Quinones et al., 2018), and other factors. Most measurement methods are scale-based, and the research on scale design and index optimization is gradually increasing, which has become one of the current research hotspots.

To sum up, it can be seen from the analysis of domain classification and topic correlation that user experience is involved in computer science, design, engineering, psychology, and other fields and has an apparent interdisciplinary nature. Among them, computer science is the leading research field, quantitative method innovation is the main research topic, and it is progressively altering the trend of big data and customization.

## Research hotspots exploration

### Research topic clustering

**FIGURE 6:**
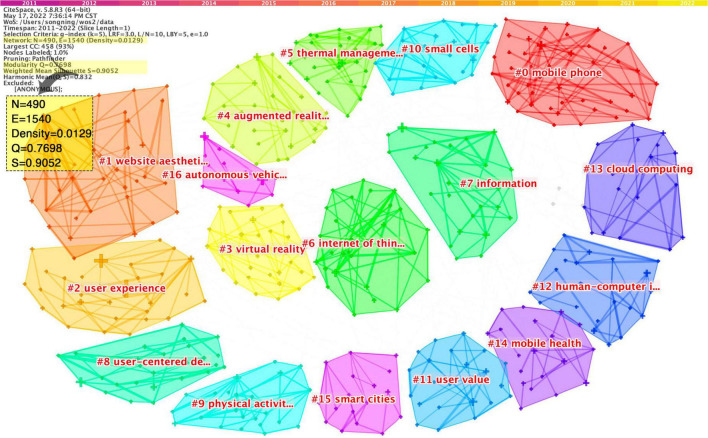
Keywords clustering network.

Research topic clustering aims to explore hot issues in the research field by analyzing the keywords with high frequency and strong centrality and revealing the deconstruction of discipline knowledge and research paradigm from the macro-level (Chen and Song, 2019). Keywords are the core and essence of an article, are a strong summary of the article’s topic, and accurately condense the author’s research direction. In order to explore the research characteristics and development context of user experience research, this paper performs keyword clustering on the literature from 2010 to 2022 and sets the clustering node attribute to “Keyword.” The results are illustrated in Figure 6.

Figure 6 contains a total of 17 clusters, consisting of 490 nodes and 1,540 connections; the network density is 0.0129, the *Q*-value is 0.7698, and the *S*-value is 0.9052, showing a good clustering effect. The clustering topics and keywords in the figure are exported and sorted separately, as shown in Table 4. For ease of interpretation, replace #6 Internet of Things in Figure 6 with the same keyword, “Artificial Intelligence.”

**TABLE 4:** Keywords clustering network analysis.

ID	Topic	Cluster keywords	Size	Year
0	Mobile phone	Mobile phone (17.61, 1.0E-4); mobile apps (9.83, 0.005); user requirements (8.38, 0.005); digital health (8.38, 0.005); smart phone (8.21, 0.005)	47	2016
1	Website aesthetics	Website aesthetics (11.83, 0.001); quality of experience (10.87, 0.001); user studies (8.12, 0.005); preference (6.5, 0.05); resource allocation (6.5, 0.05)	39	2015
2	User experience	User experience (90.96, 1.0E-4); usability (17.9, 1.0E-4); elderly (12.02, 0.001); 5 g mobile communication (12.02, 0.001); machine learning (9.04, 0.005)	33	2016
3	Virtual reality	Virtual reality (vr) (17.23, 1.0E-4); service design (7.47, 0.01); authority control (7, 0.01); business models (7, 0.01); human-machine interaction (hmi) (7, 0.01)	29	2017
4	Augmented reality	Augmented reality (40.61, 1.0E-4); human factors (11.29, 0.001); product design (9.25, 0.005); design practice (8.81, 0.005); environment (8.81, 0.005)	28	2016
5	Thermal management	Thermal management (11.14, 0.001); deep learning (11.14, 0.001); estimation (11.14, 0.001); serious games (10.57, 0.005); children (8.78, 0.005)	28	2016
6	Artificial intelligence	Internet of things (28.33, 1.0E-4); artificial intelligence (17.95, 1.0E-4); quality of service (11.94, 0.001); cloud manufacturing (11.25, 0.001); smart home (10.41, 0.005)	28	2016
7	Information	Information (14.66, 0.001); experimental evaluation (10.08, 0.005); embodied cognition (10.08, 0.005); privacy (10.08, 0.005); engagement (8.73, 0.005)	27	2014
8	User-centered design	User-centered design (17.71, 1.0E-4); user experience design (16.06, 1.0E-4); virtual environments (15, 0.001); scrum (10.7, 0.005); human-centered design (9.82, 0.005)	27	2015
9	Physical activity	Physical activity (18.29, 1.0E-4); participatory design (12.08, 0.001); patient portal (8.36, 0.005); health informatics (8.36, 0.005); older adults (6.73, 0.01)	26	2016
10	Small cells	Small cells (18.8, 1.0E-4); user experience (16.59, 1.0E-4); impact (14.39, 0.001); resource management (14.39, 0.001); energy efficiency (12.52, 0.001)	24	2015
11	User value	User value (12.77, 0.001); electronic health record (12.61, 0.001); patient education (9.04, 0.005); interface design (7.4, 0.01); ambient assisted living (7.4, 0.01)	22	2014
12	Human-computer interaction	Human-computer interaction (20.85, 1.0E-4); human computer interaction (18.91, 1.0E-4); task analysis (13.2, 0.001); human-robot interaction (12.43, 0.001); user interfaces (11.26, 0.001)	22	2017
13	Cloud computing	Cloud computing (31.74, 1.0E-4); edge computing (29.93, 1.0E-4); user experience (13.57, 0.001); neural network (13.01, 0.001); base stations (9.3, 0.005)	22	2016
14	Mobile health	Mobile health (18.25, 1.0E-4); secondary prevention (11.36, 0.001); virtualization (11.36, 0.001); mobile applications (10.98, 0.001); health (7.66, 0.01)	22	2015
15	Smart cities	Smart cities (15.03, 0.001); motivation (10.67, 0.005); flow (8.86, 0.005); human immersion (7.72, 0.01); sustainable consumption (7.72, 0.01)	17	2016
16	Autonomous vehicles	Virtual reality (53.67, 1.0E-4); autonomous vehicles (10.39, 0.005); industry 4 (6.73, 0.01)	13	2014

**FIGURE 7:**
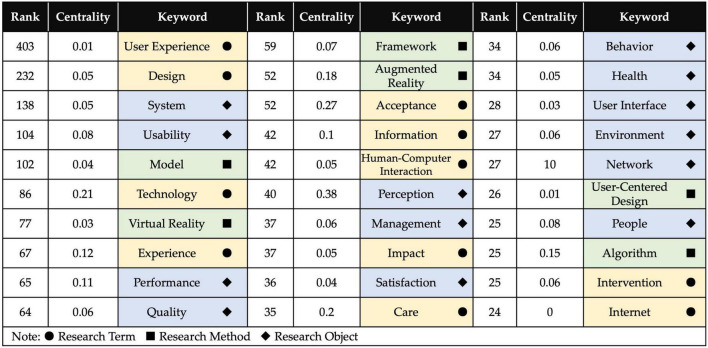
Keywords attribute classification.

In order to enhance the rationality and credibility of the research topic clustering, the top 30 keywords were extracted by frequency. According to the three dimensions of research term, research method, and research object, the classification of keyword attributes is completed, as shown in Figure 7.

By comprehensively analyzing the information in Figures 6, 7 and Table 4, we divided the 17 clusters into three directions: mobile interaction, Internet technology, and human health. The following is an analysis of the research hotspots of user experience from these three directions.

1. **Mobile interaction and user experience**

Under big data background, mobile interaction has become the main content and research hotspot of user experience research. Clusters #0 Mobile Phone, #1 Website Aesthetics, #7 Information, and #12 Human-Computer Interaction all belong to the category of mobile interaction research. The keywords involved mainly revolve around electronic device interaction design and interface evaluation, such as user needs, smart phones, quality of experience, user research, experimental evaluation, embodied cognition, task analysis, and user interface. Therefore, user satisfaction analysis and interface usability testing have become hot issues in user experience research. How to design experiments for target interfaces or interactive functions and determine experimental elements and evaluation criteria are the focus and difficulty of user experience research in mobile interaction.

2. **Internet technology and user experience**

The bidirectional application of Internet technology and user experience is another hotspot in current user experience research. On the one hand, clustering #3 Virtual Reality, #4 Augmented Reality, and #13 Cloud Computing represent the application of Internet technology in user experience research. Corresponding to virtual reality, augmented reality, service design, edge computing, neural network, permission control, and other high-frequency keywords. It shows that the Internet relies on big data and cloud computing technologies to assist in user demand capture and behavior analysis tasks. On the other hand, clusters #5 Thermal Management, #6 Artificial Intelligence, #10 Small Cells, and #16 Autonomous Vehicles represent user experience research applications in Internet technology development. It corresponds to deep learning, artificial intelligence, quality of service, smart home, resource management, and other keywords. It shows that optimizing and upgrading user experience has become one of the breakthroughs in the innovation and iteration of computer control theory and algorithm. Moreover, it is the integration of the design discipline and the computer discipline.

3. **Human health and user experience**

Attention to human health issues is another hotspot of user experience research in the era of big data. Clusters #2 User Experience, #8 User-Centered Design, #9 Physical Activity, #11 User Value, #14 Mobile Health, and #15 Smart Cities are all related to human health issues, and the keywords involved can be divided into two categories. The first is user research based on the elderly group, such as keyword availability, sports activities, user value, patient education, motivation, and immersion experience. It analyzes users’ perceived preferences, behavioral feedback, and emotional feelings toward products or services. The second is a scenario study focusing on the living environment. Keywords include participatory design, virtualization, mobile applications, and smart cities, focusing on the interaction between healthy environments and people. In addition, user experience design has expanded to other subjects, such as human lifestyles, consumption concepts, and environmental awareness. Moreover, it has also received significant attention in social innovation and service design.

### Evolution of research methods

**FIGURE 8:**
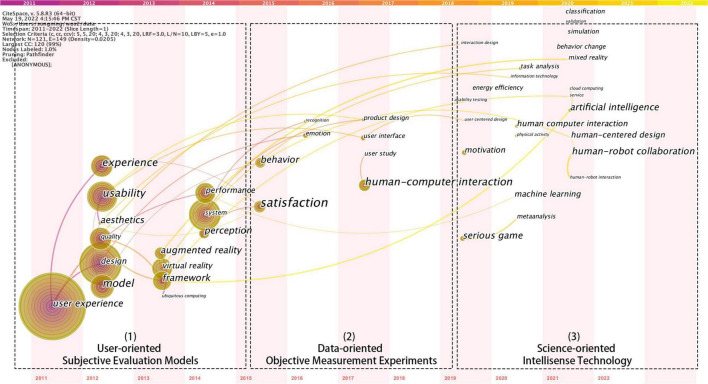
Timeline map of research method keywords.

User experience involves various disciplines and research contents, including multiple research perspectives such as users, products or services, and interactive environments, which leads to the diversity and intersection of user experience research methods. In order to reasonably divide the research methods and analyze their evolution paths, we clustered the keywords and then screened them for a second time, only the words related to the research methods are retained, and the timeline map of the research methods is generated. Combining the changes in years and the attributes of keywords, the user experience research methods are divided into three categories: user-oriented subjective evaluation models, data-oriented objective measurement experiments, and science-oriented intellisense technology, as shown in Figure 8.

1. **User-oriented subjective evaluation method (2011–2015)**

Most of the research on user-oriented subjective evaluation methods appeared in 2011–2015 and analyzed issues such as user experience concepts, theoretical frameworks, and evaluation factors from a macro perspective. Among them, conventional methods include the Kano model (Borgianni and Rotini, 2015), AHP (Liu et al., 2018), user interview method (Olsson et al., 2013), subjective evaluation scale (Tourancheau et al., 2012), and other research methods. In addition, some scholars provide a reference for user experience optimization and iteration from different perspectives such as product experience, interaction situation, and psychological needs based on experience elements, research content, and analysis perspectives. For example, Kujala et al. (2011) proposed a “UX curve” model to detect the trend of product attractiveness and the change in user satisfaction through the research on the interaction between users and products, to help enterprises improve product satisfaction and user stickiness. Partala and Kallinen (2012) analyzed the three dimensions of user emotional experience, psychological needs, and situational factors, used the PANAS emotional scale to obtain user emotional experience, and proposed the necessity and inadequacy of entertainment and social factors in user experience research. Saariluoma (2020) constructed human-computer interaction research models based on psychological theories and methods, focusing on the basic emotions and psychological experiences involved in user experience, and proposed a “bipolar ability frustration model” to help researchers analyze shifts in user sentiment.

2. **Data-oriented objective measurement experiment (2016–2019)**

Compared with the subjective evaluation model, the experimental instrument can collect and measure more objective user data to realize the quantitative evaluation of user experience research. As seen in Figure 8, from 2016 to 2019, the research on objective measurement experiments gradually increased. The user experience research method gradually shifted from subjective evaluation to data analysis combining subjective and objective. Moreover, the research objects mainly were specific products or industries fields. For example, Ko et al. (2019) proposed a user experience model with the help of virtual reality technology and quantified the user experience information in the virtual space by studying the environmental space, user activities, and service objects. Burger et al. (2018) evaluated the applicability of eye-tracking devices in user experience evaluation and thus completed the evaluation of mobile application software. Kim et al. (2020) proposed a pseudo-tactile interface research method for optimizing a products haptic system, using myoelectric sensors to analyze the user’s grip force, and generating an immersive virtual application to investigate and analyze the usage of the product usage. Shim and Lee (2015) proposed an enhanced algorithm through the analysis of EMG data, using deep belief network technology to identify multi-channel EMG patterns to optimize the computer interface system.

3. **Science-oriented intellisense technology (2020-2022)**

Due to environmental influences and breakthroughs in artificial intelligence technology, user experience research methods have gradually changed in the post-epidemic era. It transitions from an objective measurement experiment to an intelligent perception technology that integrates multi-dimensional information such as user emotions, physiological needs, and emotional perception. Moreover, virtual scenes are constructed based on multi-dimensional data fusion to obtain a more realistic user experience. As can be seen from Figure 8, keywords such as “Human-Robot Collaboration,” “Artificial Intelligence,” and “Machine Learning” have gradually become hot research technologies around 2020, which can be divided into two directions: virtual reality enhancement and user demand prediction. Research on virtual reality augmentation technology mainly focuses on environment simulation and research tool optimization. For example, Jeong et al. (2020) proposed an asymmetric interface to provide a more realistic sense of presence for both head-mounted and ordinary display users. They provide an experience consistent with the user’s environment in asymmetric virtual reality. Yi and Kim (2021) used wearable mixed reality technology as a research tool with the help of user experience testing, interactive questionnaire research, website aesthetic testing, and other methods. The experience is evaluated from multiple perspectives to provide recommendations for applying wearable mixed reality technology to user experience.

Furthermore, the research on user demand prediction technology mainly relies on computer algorithms and models to mine user information and realize the evaluation of user experience. For example, Shin (2020, 2022) proposed to use AI algorithms to obtain user perception and use artificial intelligence technology to create immersive user experience. Yeon et al. (2020) took intelligent speakers as the research goal, used text mining technology to mine user comment datasets, and proposed a method to enhance the user experience of intelligent speakers. Pandiyarajan and Shanmugavadivel (2022) based on the Markov chain Monte Carlo square technology and used image processing methods to mine user evaluation opinions. They studied the emotional state of user experience data set evaluation through the emotional characteristics involved in user experience design.

## Future trend forecast

**FIGURE 9:**
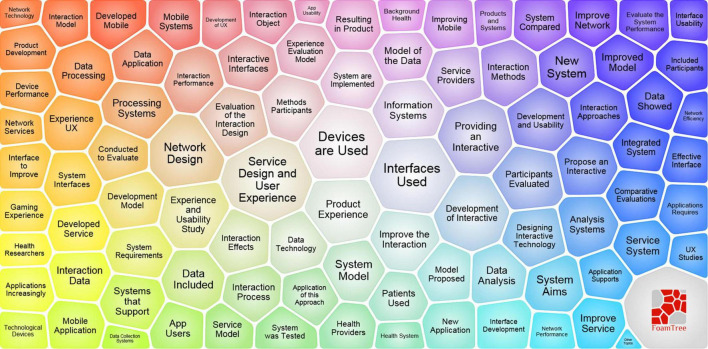
Future topic bubble map.

Future trend forecast refers to the integration and analysis of recent research themes or themes that have appeared in the past but become research hotspots shortly and obtain future research directions accordingly. In order to effectively improve the efficiency of research topic acquisition and analysis, this paper uses the Lingo clustering algorithm of CARROT2 software to identify the subject headings of the documents from 2011 to 2022. Figure 9 shows the results. The size of the bubbles in the figure represents the number of documents on the subject.

According to Figure 9, there are 90 bubble themes in the figure, and the distribution and size of the bubbles are relatively uniform. It indicates that the research content of user experience is relatively scattered and has been widely used in medical and health, industrial manufacturing, intelligent computing, social services, and other fields. In order to accurately predict the research direction, the bubble chart is classified according to the subject heading attributes, focusing on the following three research directions.

1. **Immersive Experience Upgrades with Multi-scene Integration**

The “era of digital intelligence empowerment” is about to enter the “metaverse era,” and the process of social virtualization continues to accelerate. The development of technologies such as virtual reality, augmented reality, digital twin, and artificial intelligence closely integrates virtual and natural scenes and provides a new direction for user experience research (Shen, 2022). Under the influence of the metaverse environment, the user experience will gradually shift from the authentic traditional scene and mobile application experience research to the user’s research in the virtual space and the upgrade of the virtual experience, which can be carried out from the following three aspects.

1. **The user experience content that combines virtual and real.** Under the metaverse form and technological empowerment, users gradually accept contactless services, and the evaluation and optimization of product and system user experience will gradually be extended to online, forming a situation where offline and online are integrated.
2. **The user experience process of timely feedback.** In the process of social virtualization, user experience is being combined with technologies such as virtual reality, platform architecture, and terminal equipment. Through resource integration and digital transformation, users can obtain the physiological and psychological feelings on the virtual platform and try to provide users with cross-space, cross-region, and cross-scenario service content.
3. **The decentralized user experience object.** Presently, user experience research is aimed chiefly at disadvantaged groups such as children and the elderly. The focus on shared experience and the data capture mode needs to be improved. The concept of decentralization and equalization will provide suggestions for selecting user experience research objects.

Based on the themes of “Gaming Experience,” “Network Design,” and “Service Model” in Figure 9, how to connect users’ physical experience with virtual experience, how to weaken the sense of spatial connection, and improve service fluency will be one of the critical points of future user experience research.

2. **Multi-role Collaboration Innovative Design of Crowd Intelligence**

In the context of big data, increasingly complex design scenarios and user needs put higher requirements on the research scope and design accuracy of user experience design. With its co-creation, intelligence, and fault tolerance, swarm intelligence design provides a new idea of multi-role, multi-level and collaborative research for user experience design (Zhang et al., 2018). Compared with the traditional user-centered research on user experience, the user experience of collective intelligence co-creation is based on the participation modes and user experience types of all parties in the research process. The network platform is used to carry out resource sharing and value co-creation, aiming to develop research processes and multi-role virtual collaboration cooperation scenarios. According to the topics of “Methods Participants,” “App Users,” “Service Providers,” and “Health Providers” in Figure 9, the user experience guided by quorum innovation should include multiple roles such as designers, users, and service providers. From the two aspects of information collection and experience optimization.

1. **Multi-terminal collaborative information collection.** The client can track, collect, and upload user information of different roles with the help of smart devices or mobile platforms. At the same time, the product side collects data such as user preferences, living habits, and operating procedures based on user usage. The environmental side collects information such as geographic location, temperature, and humidity through GPS positioning, scene monitoring, satellite signals, and other equipment.
2. **Dynamic, customized experience optimization.** Designers will build user models through information collection results and match personalized services according to user needs so that user experience design tends to be precise and customized. At the same time, it can further expand the application field of user experience and transition from product or service experience design to life experience design.

3. **Cross-disciplinary interactive exploration of multi-disciplinary**

The development of artificial intelligence and Internet of Things technology has gradually integrated intelligent hardware such as natural user interfaces, tangible media, and new interaction paradigms (Joly et al., 2019). From the themes of “Data Technology,” “Model of Data,” and “Designing Interactive Technology,” user experience has been continuously expanded in the fields of computer, mathematical statistics, and psychology. Moreover, it is applied to algorithm optimization, data mining, emotion recognition, and other fields. This shows the trend of integration of design research and science and technology; multi-disciplinary and interdisciplinary research will become another direction for the future development of user experience.

1. **User experience guides the development of science and technology and provides direction for algorithm analysis and model building.** User experience brings user needs into algorithm design. By simulating user behavior habits and thinking methods, the mobile platform can achieve the design goals of more reasonable interaction logic, clearer functions, and more exciting operations.
2. **Science and technology provide back-end support for user experience research.** The themes of life, health, and smart cities will become an emerging trend in user experience design. The differentiation, acquisition, and integration of user data at different levels, such as the perception layer, network layer, information layer, and interaction layer, as well as the orientation of online medical care, public lifestyles, social modes, and interactive behaviors, will all become difficult points in user experience research.

## Future trend forecast

**FIGURE 10:**
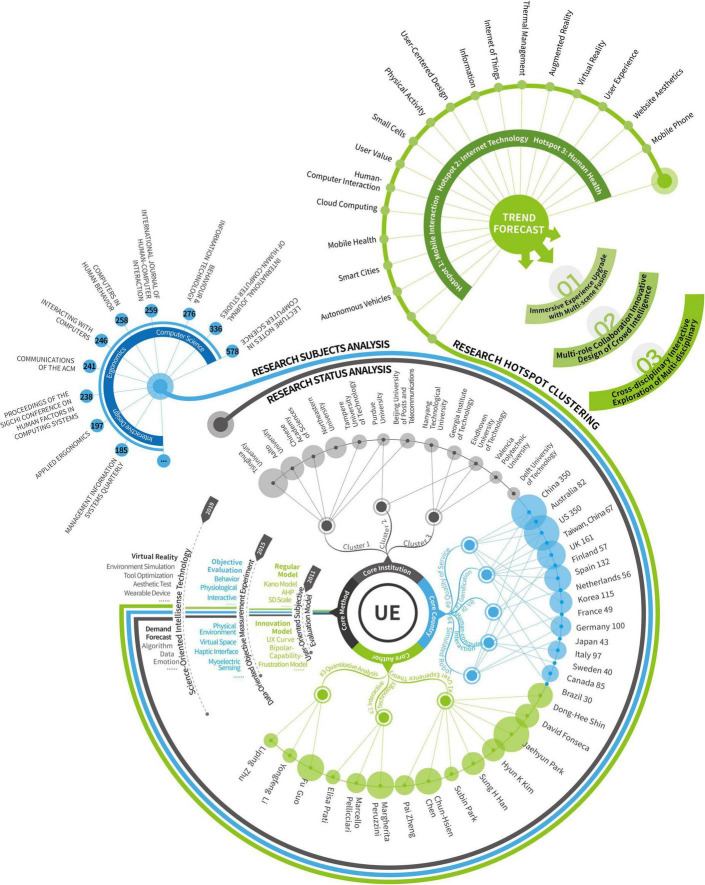
User experience knowledge map.

The knowledge map is a graph showing the development process and trend prediction of scientific knowledge. Constructing a knowledge map can clearly show and summarize the discipline’s current development status and hot trends. Based on the above research results, the user experience’s knowledge map is constructed from three levels of research status, research subjects, and research hotspots, as shown in Figure 10.

Figure 10 contains three information rings. Among them, the inner gray information ring is the research status analysis. The information integration and clustering of the research status of user experience are carried out from the four themes of core institutions, core countries, core authors, and core methods. The middle-level blue information ring represents the core research themes. According to the analysis of the core journals, the user experience is divided into three directions: interaction design-centered, ergonomics-centered, and computer science-centered. The outer green information ring represents the clustering of research hotspots. We predict user experience research hotspots in this area and explore future research trends through keyword analysis.

## Conclusion

This paper uses the Web of Science core collection database as the data source. It selects 1,759 pieces of literature from January 2011 to April 2022 to explore the research status and hotspots of user experience. Furthermore, it combines the analysis results to predict the future development direction and build a knowledge map. In order to provide a new perspective for user experience research. The main conclusions are as follows:

1. **General research trend:** During the data statistics period, the research field of user experience has gradually expanded. On the one hand, it has flourished in computer science, systematics, and psychology. On the other hand, it is constantly advancing in emerging fields such as life sciences, metaverse, and virtual technology.
2. **Research strength:** User experience research is involved in many institutions and countries, but there are differences in research focus. Among them, in Asia, mainly China, the research topics primarily focus on theoretical research on user experience, quality evaluation, user perception measurement, and network information security. While the United States and other related countries mainly explore the collaborative application of computer technology and user experience. At the same time, the distribution of core authors is relatively scattered, and there is a lack of comparative and in-depth collaborative research on specific topics.
3. **Research subjects:** User experience research is mainly distributed in three subjects: interaction design, ergonomics, and computer science. In theme development, in the past 10 years, user experience research has experienced a path change from concept definition to macro-framework and model research, to micro-tool and method innovation, with prominent interdisciplinary characteristics.
4. **Research hotspots:** User experience keywords can be clustered into three research hotspots, mobile interaction, Internet technology, and human health, and in-depth research and exploration of user experience are carried out from the three fields of systems, technology, and humanities. At the same time, the research method has also changed with the changes in time and hot spots. Furthermore, we divided it into three stages: “user-oriented subjective evaluation model,” “data-oriented objective measurement experiment,” “and “science-oriented intelligent perception technology.” It shows that the user experience changes from qualitative to quantitative to the research process and development context of qualitative and quantitative fusion.
5. According to the analysis results of research strength, research topics and hotspots, combined with the topic bubble map, predict the future development trend and build a complete knowledge map. Under the background of big data, artificial intelligence, metaverse, and other themes, the future user experience may focus on three research directions: “immersive experience upgrades with multi-scene integration,” “multi-role collaboration and crowd-intelligence innovation design,” and “multi-disciplinary cross-domain interactive exploration.” It will assist with research on virtual reality scene construction, life behavior guidance, and data algorithm optimization.

It should be noted that due to the limitation of the retrieval database, there are certain missing data sets selected for the study. Future research can enrich data and analyze user experience more comprehensively, such as supplementing domestic literature, national funds, and patented technologies, to analyze user experience research trends and disciplinary connotations comprehensively and deeply.

## Data availability statement

The original contributions presented in this study are included in the article/supplementary material, further inquiries can be directed to the corresponding author.

## Author contributions

XH provided the research idea and the purpose of this research, supervised, corrected, and revised this manuscript. YK prepared the original draft. NS designed the study, analyzed the data, and wrote the initial draft preparation. All authors have read and agreed to the published version of the manuscript.

## References

[B1] Adikari S. McDonald C. Campbell J. (2011). “A Design Science Framework for Designing and Assessing User Experience,” in *International Conference on Ergonomics and Health Aspects of Work with Computers (EHAWC)/14th International Conference on Human-Computer Interaction (HCI)*) (Berlin: Springer), 25–34. 10.1007/978-3-642-21602-2_3

[B2] Bitkina O. V. Kim H. K. Park J. (2020). Usability and user experience of medical devices: An overview of the current state, analysis methodologies, and future challenges. *Int. J. Indust. Ergon.* 76:102932. 10.1016/j.ergon.2020.102932

[B3] Borgianni Y. Rotini F. (2015). Towards the fine-tuning of a predictive Kano model for supporting product and service design. *Total Qual. Manag. Bus. Excell.* 26 263–283. 10.1080/14783363.2013.791119

[B4] Burger G. Guna J. Pogacnik M. (2018). Suitability of Inexpensive Eye-Tracking Device for User Experience Evaluations. *Sensors* 18:1822. 10.3390/s18061822 PMC6022140 29874794

[B5] Chen C. M. Song M. (2019). Visualizing a field of research: A methodology of systematic scientometric reviews. *PLoS One* 14:e0223994. 10.1371/journal.pone.0223994 31671124 PMC6822756

[B6] Cho Y. Park J. Han S. H. Kang S. (2011). Development of a web-based survey system for evaluating affective satisfaction. *Int. J. Indust. Ergon.* 41 247–254. 10.1016/j.ergon.2011.01.009

[B7] Desmet P. Hekkert P. (2007). Framework of Product Experience. *Int. J. Design* 1 57–66. 10.1016/B978-008045089-6.50003-4

[B8] Ding Y. Guo F. Zhang X. Qu Q. Liu W. (2016). Using event related potentials to identify a user’s behavioural intention aroused by product form design. *Appl. Ergon.* 55 117–123. 10.1016/j.apergo.2016.01.018 26995041

[B9] Feng L. Wei W. (2019). An Empirical Study on User Experience Evaluation and Identification of Critical UX Issues. *Sustainability* 11:2432. 10.3390/su11082432

[B10] Fong J. S. Ching L. L. Chiat K. C. Malim N. H. A. H. Husin M. H. Singh M. M. (2015). “Localizing User Experience For Mobile Application: A Case Study Among Usm Undergraduates,” in *5th International Conference on Computing & Informatics*,(Istanbul) 366–373.

[B11] Guo F. Ding Y. Liu W. L. Liu C. Zhang X. F. (2016). Can eye-tracking data be measured to assess product design?: Visual attention mechanism should be considered. *Int. J. Indust. Ergon.* 53 229–235. 10.1016/j.ergon.2015.12.001

[B12] Guo H. (2016). “Lean but not Mean UX: Towards a Spiral UX Design Model,” in *Design, User Experience, and Usability: Design Thinking and Methods*, ed. Marcus A. (Berlin: Springer International Publishing), 25–33.

[B13] Haryaka U. Agus F. Kridalaksana A. H. (2017). User satisfaction model for e-learning using smartphone. *Proc. Comput. Sci.* 116 373–380. 10.1016/j.procs.2017.10.070

[B14] Hassenzahl M. Diefenbach S. Goritz A. (2010). Needs, affect, and interactive products - Facets of user experience. *Interact. Comput.* 22 353–362. 10.1016/j.intcom.2010.04.002

[B15] Hou J. Yang X. Chen C. (2018). Emerging trends and new developments in information science: A document co-citation analysis (2009–2016). *Scientometrics* 115 869–892. 10.1007/s11192-018-2695-9

[B16] Huang Y. Chen C.-H. Khoo L. P. (2012). Kansei clustering for emotional design using a combined design structure matrix. *Int. J. Indust. Ergon.* 42 416–427. 10.1016/j.ergon.2012.05.003

[B17] Jeong K. Kim J. Kim M. Lee J. Kim C. (2020). Asymmetric Interface: User interface of asymmetric virtual reality for new presence and experience. *Symmetry Basel* 12:53. 10.3390/sym12010053

[B18] Joly M. P. Teixeira J. G. Patrício L. Sangiorgi D. (2019). Leveraging service design as a multidisciplinary approach to service innovation. *J. Serv. Manag.* 30 681–715. 10.1108/JOSM-07-2017-0178

[B19] Karray F. Alemzadeh M. Abou Saleh J. Arab M. N. (2017). Human-computer interaction: Overview on state of the art. *Int. J. Smart Sens. intell. Syst.* 1 137–159. 10.21307/ijssis-2017-283

[B20] Kim M. Kim J. Jeong K. Kim C. (2020). Grasping VR: Presence of pseudo-haptic interface based portable hand grip system in immersive virtual reality. *Int. J. Hum. Comput. Interact.* 36 685–698. 10.1080/10447318.2019.1680920

[B21] Ko I. Kim D. Park J. H. (2019). A user experience environment model for human activity simulation. *Futur. Gene. Comput. Syst. Int. J. Esci.* 96 660–666. 10.1016/j.future.2017.07.064

[B22] Kujala S. Roto V. Vaananen-Vainio-Mattila K. Karapanos E. Sinnela A. (2011). UX Curve: A method for evaluating long-term user experience. *Interact. Comput.* 23 473–483. 10.1016/j.intcom.2011.06.005

[B23] Law E. L. C. Roto V. Hassenzahl M. Vermeeren A. Kort J. (2009). “Understanding, Scoping and Defining User eXperience: A Survey Approach,” in *27th Annual CHI Conference on Human Factors in Computing Systems*, (Boston, MA) 719–728. 10.1145/1518701.1518813

[B69] Law E. L. C. van Schaik P. (2010). Modeling user experience – an agenda for research and practice. *Interact. Comput*. 22, 313–322. 10.1016/j.intcom.2010.04.006

[B70] Law E. L. -C. Van Schaik P. Roto V. (2014). Attitudes towards user experience (UX) measurement. *Int. J. Hum. Comput. Stud.* 72, 526–541.

[B24] Li R. Zhang H. Liu C. Qian Z. C. Zhang L. (2022). Bibliometric and Visualized Analysis of User Experience Design Research: From 1999 to 2019. *SAGE Open* 12:21582440221. 10.1177/21582440221087266

[B25] Li Y. F. Zhu L. P. (2017). Optimisation of product form design using fuzzy integral-based Taguchi method. *J. Eng. Design* 28 480–504. 10.1080/09544828.2017.1346239

[B26] Li Y. F. Zhu L. P. (2019). Optimization of user experience in interaction design through a Taguchi-based hybrid approach. *Hum. Factors Ergon. Manuf. Serv. Indust.* 29 126–140. 10.1002/hfm.20765

[B27] Lin S. Shen T. Guo W. (2021). Evolution and emerging trends of kansei engineering: A Visual Analysis Based on CiteSpace. *IEEE Access* 9 111181–111202. 10.1109/ACCESS.2021.3102606

[B28] Liu J. Cui M. Jia L. (2018). “Evaluation of user experience based 3D websites using gray correlation analysis and AHP,” in *2018 14th International Conference on Natural Computation, Fuzzy Systems and Knowledge Discovery (ICNC-FSKD)*, (Piscataway, NJ), 1306–1309. 10.1109/FSKD.2018.8687167

[B29] Olsson T. Lagerstam E. Kärkkäinen T. Väänänen-Vainio-Mattila K. (2013). Expected user experience of mobile augmented reality services: A user study in the context of shopping centres. *Pers. Ubiquitous Comput.* 17 287–304. 10.1007/s00779-011-0494-x

[B30] Pandiyarajan R. Shanmugavadivel K. (2022). Opinion mining for user experience evaluation model using Bayesian estimation of Markov Chain Monte Carlo technique. *Dyna* 97, 189–194. 10.6036/10303

[B31] Park J. Han S. H. (2013). Defining user value: A case study of a smartphone. *Int. J. Indust. Ergon.* 43 274–282. 10.1016/j.ergon.2013.04.005

[B32] Park J. Han S. H. Kim H. K. Oh S. Moon H. (2013). Modeling user experience: A case study on a mobile device. *Int. J. Indust. Ergon.* 43 187–196. 10.1016/j.ergon.2013.01.005

[B33] Partala T. Kallinen A. (2012). Understanding the most satisfying and unsatisfying user experiences: Emotions, psychological needs, and context. *Interact. Comput.* 24 25–34. 10.1016/j.intcom.2011.10.001

[B34] Peruzzini M. Germani M. Marilungo E. (2013). “Design for sustainability of product-service systems in the extended enterprise,” in *20th ISPE International Conference on Concurrent Engineering*, (Melbourne) 314–323.

[B35] Peruzzini M. Grandi F. Pellicciari M. (2020). Exploring the potential of Operator 4.0 interface and monitoring. *Comput. Indust. Eng.* 139:105600. 10.1016/j.cie.2018.12.047

[B71] Porat T. Tractinsky N. (2012). It’s a pleasure buying here: the effects of web-store design on consumers’ emotions and attitudes. *Hum. Comput. Interact*. 27, 235–276. 10.1080/07370024.2011.646927

[B72] Preece H. S. J. Rogers R. (2015). *Interaction Design: Beyond Human-computer Interaction*. New York: John Wiley and Sons.

[B36] Quinones D. Rusu C. Rusu V. (2018). A methodology to develop usability/user experience heuristics. *Comput. Stand. Interf.* 59 109–129. 10.1016/j.csi.2018.03.002

[B37] Rawat K. S. Sood S. K. (2021). Knowledge mapping of computer applications in education using CiteSpace. *Comput. Appl. Eng. Educ.* 29 1324–1339. 10.1002/cae.22388

[B38] Saariluoma P. (2020). “User psychology of emotional interaction—usability, user experience and technology ethics,” in *Emotions in Technology Design: from Experience to Ethics*, (Berlin: Springer), 15–26. 10.1007/978-3-030-53483-7_2

[B73] Schrepp M. Hinderks A. Thomaschewski J. (2017a). Construction of a benchmark for the user experience questionnaire (UEQ). *Int. J. Interact. Multi. Artific. Intell.* 4, 40–44. 10.9781/ijimai.2017.445 34887180

[B74] Schrepp M. Hinderks A. Thomaschewski J. (2017b). Design and evaluation of a short version of the user experience questionnaire (UEQ-S). *Int. J. Interact. Multi. Artific. Intell.* 4, 103–108. 10.9781/ijimai.2017.09.001 34887180

[B39] Seo K. K. Lee S. Chung B. D. Park C. (2015). Users’ Emotional Valence, Arousal, and Engagement Based on Perceived Usability and Aesthetics for Web Sites. *Int. J. Hum. Comput. Interact.* 31 72–87. 10.1080/10447318.2014.959103

[B40] Shen R. (2022). Research on the Design of the Red Culture Digital Exhibition Hall Based on Metaverse. *Asian J. Soc. Sci. Stud.* 7:102. 10.20849/ajsss.v7i3.1046

[B41] Shengli D. (2008). The advancement of the study on foreign user experience. *Library Inf. Serv.* 52:43.

[B42] Shim H.-M. Lee S. (2015). Multi-channel electromyography pattern classification using deep belief networks for enhanced user experience. *J. Central South Univ.* 22 1801–1808. 10.1007/s11771-015-2698-0

[B43] Shin D. (2018). Empathy and embodied experience in virtual environment: To what extent can virtual reality stimulate empathy and embodied experience? *Comput. Hum. Behav.* 78 64–73. 10.1016/j.chb.2017.09.012

[B44] Shin D. (2019). How do users experience the interaction with an immersive screen? *Comput. Hum. Behav.* 98 302–310. 10.1016/j.chb.2018.11.010

[B45] Shin D. (2020). How do users interact with algorithm recommender systems? The interaction of users, algorithms, and performance. *Comput. Hum. Behav.* 109 106344. 10.1016/j.chb.2020.106344

[B46] Shin D. (2022). Expanding the role of trust in the experience of algorithmic journalism: User sensemaking of algorithmic heuristics in Korean users. *Journal. Pract.* 16 1168–1191. 10.1080/17512786.2020.1841018

[B47] Shin D. H. (2009). An empirical investigation of a modified technology acceptance model of IPTV. *Behav. Inf. Technol.* 28 361–372.

[B48] Shin D.-H. (2017). The role of affordance in the experience of virtual reality learning: Technological and affective affordances in virtual reality. *Telemat. Inf.* 34 1826–1836. 10.1016/j.tele.2017.05.013

[B49] Svezhenov Y. Nikov A. Dimitrov L. (2019). “Modelling in Dynamic User Experience Design of Educational Toys Packages,” in *45th International Conference on Application of Mathematics in Engineering and Economics (AMEE)*). (Dundee) 10.1063/1.5133592

[B50] Tourancheau S. Sjöström M. Olsson R. Persson A. Ericson T. Rudling J. (2012). “Subjective evaluation of user experience in interactive 3D visualization in a medical context,” in *Medical Imaging 2012: Image Perception, Observer Performance, and Technology Assessment*, (Bellingham), 314–326. 10.1117/12.910828

[B51] Vermeeren A. P. Law E. L.-C. Roto V. Obrist M. Hoonhout J. Väänänen-Vainio-Mattila K. (2010). “User experience evaluation methods: Current state and development needs,” in *Proceedings of the 6th Nordic conference on human-computer interaction: Extending boundaries*, (London) 521–530. 10.1145/1868914.1868973

[B52] Villagrasa S. Fonseca D. Redondo E. Duran J. (2014). Teaching case of gamification and visual technologies for education. *J. Cases Inf. Technol.* 16 38–57. 10.4018/jcit.2014100104

[B53] Wang C. Chen L. Zhao L. Li M. (2013). “Exploring the Norms for the UX Design of Intelligent Products: A Case Study,” in *IEEE Tsinghua International Design Management Symposium (TIDMS)*,(Shenzhen) 158–165. 10.1109/TIDMS.2013.6981232

[B54] Wang S. Lv X. (2021). Hot topics and evolution of frontier research in early education: A bibliometric mapping of the research literature (2001–2020). *Sustainability* 13:9216. 10.3390/su13169216

[B55] Wei F. Zhang G. (2020). A document co-citation analysis method for investigating emerging trends and new developments: a case of twenty-four leading business journals. *Inform. Res.* 25:842.

[B56] Wu H. Y. Luo W. Z. Pan N. Nan S. H. Deng Y. Y. Fu S. Q. (2019). Understanding freehand gestures: a study of freehand gestural interaction for immersive VR shopping applications. *Hum. Cent. Comput. Inf. Sci.* 9:43. 10.1186/s13673-019-0204-7

[B57] Wu H. Y. Wang J. M. Zhang X. L. (2016). User-centered gesture development in TV viewing environment. *Multimed. Tools Appl.* 75 1–28. 10.1007/s11042-014-2323-5

[B58] Xia H. Zhang S. (2013). “Visual Perceptions of Materials in Emotional User Experience Design,” in *3rd International Conference on Advanced Design and Manufacturing Engineering (ADME 2013)* (Switzerland: Trans Tech Publications Ltd.), 812–815. 10.4028/www.scientific.net/AMM.397-400.812

[B59] Xia W. Liu Z. (2022). “User Experience Research in China: A 15-Year Bibliometric Analysis,” in *International Conference on Human-Computer Interaction*, (Berlin: Springer), 295–313. 10.1007/978-3-031-05897-4_21

[B60] Xu L. Gao J. Chen L. Liang G. Feng H. (2022). Cultural Confidence on “Art & Engineering” Construction of Product Design under “New Liberal Arts”. *Comput. Intell. Neurosci.* 2022:6101368. 10.1155/2022/6101368 35586095 PMC9110127

[B61] Yang R. Wong C. W. Y. Miao X. (2021). Analysis of the trend in the knowledge of environmental responsibility research. *J. Clean. Prod.* 278:123402. 10.1016/j.jclepro.2020.123402

[B62] Yeon D. Park G. Kim H.-W. (2020). User experience analysis and management based on text mining: A smart speaker case. *Inf. Syst. Rev.* 22 77–99. 10.14329/isr.2020.22.2.077

[B63] Yi J. H. Kim H. S. (2021). User Experience Research, Experience Design, and Evaluation Methods for Museum Mixed Reality Experience. *Acm J. Comput. Cult. Heritage* 14 1–28. 10.1145/3462645

[B64] Yuqi L. Songyang L. Jing W. (2019). Application of artificial intelligence technology in product interaction design. *Packag. Eng.* 40 14–21.

[B65] Zhan J. Ma Y. Zhao D. Li Z. Tan H. Wang X. (2021). Knowledge atlas of post-traumatic epilepsy research: Based on citespace visualization analysis. *Epilepsy Res.* 178:106790. 10.1016/j.eplepsyres.2021.106790 34798493

[B66] Zhang J. Wang L. Shi L. An W. Wei W. (2018). “Study on crowd intelligence design pattern of the open innovation community,” in *2018 IEEE International Conference on Applied System Invention (ICASI)*, (Piscataway, NJ), 774–777. 10.1109/ICASI.2018.8394375

[B67] Zheng P. Lin T.-J. Chen C.-H. Xu X. (2018). A systematic design approach for service innovation of smart product-service systems. *J. Clean. Prod.* 201 657–667. 10.1016/j.jclepro.2018.08.101

[B68] Zhu J. Hua W. (2017). Visualizing the knowledge domain of sustainable development research between 1987 and 2015: A bibliometric analysis. *Scientometrics* 110 893–914. 10.1007/s11192-016-2187-8

